# Risk of Seizures in Patients with Organophosphate Poisoning: A Nationwide Population-Based Study

**DOI:** 10.3390/ijerph16173147

**Published:** 2019-08-29

**Authors:** Chieh-Sen Chuang, Kai-Wei Yang, Chia-Ming Yen, Cheng-Li Lin, Chia-Hung Kao

**Affiliations:** 1 Department of Neurology, Changhua Christian Hospital, Changhua 50006, Taiwan; 2 Department of Emergency, China Medical University Hospital, Taichung 40447, Taiwan; 3 Department of Anesthesiology, Buddhist Tzu Chi General Hospital, Taichung 40447, Taiwan; 4 Department of Graduate Institute of Acupuncture Science, China Medical University, Taichung 40447, Taiwan; 5 Management Office for Health Data, China Medical University Hospital, Taichung 40447, Taiwan; 6 College of Medicine, China Medical University, Taichung 40447, Taiwan; 7 Graduate Institute of Biomedical Sciences and School of Medicine, College of Medicine, China Medical University, Taichung 40447, Taiwan; 8 Department of Nuclear Medicine and PET Center, China Medical University Hospital, Taichung 40447, Taiwan; 9 Department of Bioinformatics and Medical Engineering, Asia University, Taichung 41354, Taiwan; 10 Center of Augmented Intelligence in Healthcare, China Medical University Hospital, Taichung 40447, Taiwan

**Keywords:** organophosphate poisoning, seizure disorder, retrospective cohort study

## Abstract

Objective: Previous research has demonstrated that patients with a history of organophosphate poisoning tend to have a higher risk of neurological disorder. However, research on the rate of seizure development in patients after organophosphate poisoning is lacking. This study examined whether individuals with organophosphate poisoning have an increased risk of seizures through several years of follow-up. Patients and Methods: We conducted a retrospective study on a cohort of 45,060 individuals (9012 patients with a history of organophosphate poisoning and 36,048 controls) selected from the Taiwan National Health Insurance Research Database. The individuals were observed for a maximum of 12 years to determine the rate of new-onset seizure disorder. We selected a comparison cohort from the general population that was randomly frequency-matched by age, sex, and index year and further analyzed the risk of seizures using a Cox regression model adjusted for sex, age, and comorbidities. Results: During the study period, the risk of seizure development was 3.57 times greater in patients with organophosphate poisoning compared with individuals without, after adjustments for age, sex, and comorbidities. The absolute incidence of seizures was highest in individuals aged 20 to 34 years in both cohorts (adjusted hazard ratio = 13.0, 95% confidence interval = 5.40−31.4). A significantly higher seizure risk was also observed in patients with organophosphate poisoning and comorbidities other than cirrhosis. Conclusions: This nationwide retrospective cohort study demonstrates that seizure risk is significantly increased in patients with organophosphate poisoning compared with the general population.

## 1. Introduction

Most patients exposed to organophosphates came into contact with insecticides. Some examples of organophosphate pesticides are malathion, parathion, and diazinon. Other examples of organophosphate anticholinesterases include the chemical weapon sarin and VX nerve agent (venomous agent X). Organophosphates are potent cholinesterase inhibitors capable of causing severe cholinergic toxicity following cutaneous exposure, inhalation, or ingestion. Organophosphates provoke the irreversible inhibition of acetylcholinesterase, leading to acetylcholine accumulation in muscarinic and nicotinic cholinergic synapses in the nervous system. The clinical features of organophosphate poisoning include increased saliva and tear production, diarrhea, nausea, vomiting, small pupils, sweating, muscle tremors, and confusion [1]. Approximately 10 to 40% of patients with organophosphate poisoning develop neurological disorders 1 to 4 days after exposure. The neurological symptoms include neck flexion weakness, decreased deep tendon reflexes, cranial nerve abnormalities, proximal muscle weakness, and respiratory insufficiency [2].

Clinically, respiratory failure is the main cause of death in severe organophosphate poisoning. Respiratory failure is attributed to a combination of bronchoconstriction, respiratory muscle paralysis, and damage to the medullary respiratory centers [3]. The effects of organophosphates on the central nervous system include irritability, restlessness, disorientation, and confusion, which can evolve into generalized seizures, status epilepticus, and brain damage [4]. Status epilepticus following organophosphate exposure can last 30 min or longer, causing profound brain damage resulting in neuronal damage or death [5,6,7]. The effects of organophosphate intoxication are long-lasting, and survivors experience chronic brain damage and face the risk of neurological and cognitive deficits [8,9]. Status epilepticus and chronic brain damage following organophosphate exposure can contribute to epileptogenesis [10,11]. However, no study has evaluated the rate of seizure disorder development after organophosphate poisoning using long-term follow-ups.

We used representative Taiwan National Health Insurance (NHI) data sets to form a cohort and examine whether individuals face an increased risk of seizures years after organophosphate poisoning. To date, this is the largest cohort used to investigate this topic.

## 2. Methods

### 2.1. Data Source

The present study was conducted using the Taiwan National Health Insurance Research Database (NHIRD). The NHI program was implemented in Taiwan on 1 March 1995. This program covers over 99% of the 23.74 million Taiwanese residents (http://www.nhi.gov.tw/english/index.aspx). In 1999, as part of the NHIRD project, the Bureau of NHI began to release patient data in an electronic format for research purposes. These de-identified secondary data include all registry and claims data. In this study, we used the hospitalization claims data of all enrollees in Taiwan, including sex, birth date, dates of admission and discharge, diagnoses, operations, discharge statuses, and expenditures by admission. The diagnostic codes are based on the International Classification of Diseases, Ninth Revision, Clinical Modification (ICD-9-CM). Because the data set consists of de-identified secondary data, the study was exempted from full review by the Institutional Research Ethics Committee (CMUH104-REC2-115-CR4).

### 2.2. Sample Cohort

The study cohorts were identified from inpatient claims between 1 January 2000 and 31 December 2011; individuals diagnosed with organophosphate poisoning (ICD-CM-9 code 989.3) were considered the organophosphate poisoning cohort. The date of the first admission for organophosphate poisoning was used as the index date. We excluded patients with epilepsy (ICD-9-CM code 345) before the index date, aged less than 20 years, or with incomplete information. For each patient with organophosphate poisoning, we randomly selected four controls without the condition from among the NHI beneficiaries and used the same exclusion criteria. The controls were frequency-matched with the organophosphate poisoning cohort by age (five-year groups) and sex.

### 2.3. Outcome

We identified diagnoses of epilepsy in hospitalization records from 2000 to 2011 as the study endpoint. All of the study individuals were followed up from the index date to the endpoint, withdrawal from the NHI, or the end of 2011, whichever came first.

### 2.4. Comorbidities

A history of diabetes (ICD-9-CM code 250), hypertension (ICD-9-CM code 401–405), hyperlipidemia (ICD-9-CM code 272), head injury (ICD-9-CM codes 310.2, 800, 801, 803, 804, 850, 851, 853, 854), depression (ICD-9-CM codes 296.2, 296.3, 296.82, 300.4, 311), stroke (ICD-9-CM code 430–438), cirrhosis (ICD-9-CM code 571), coronary artery disease (CAD) (ICD-9-CM codes 410–414), congestive heart failure (CHF) (ICD-9-CM code 428), or atrial fibrillation (AF) (ICD-9-CM code 427.31) identified at baseline from the patients’ hospitalization records was considered a comorbidity.

### 2.5. Statistical Analysis

The distributions of baseline characteristics (age, sex, and comorbidities) of the organophosphate poisoning and control cohorts were compared using the chi-square test. We used the Student’s t-test for continuous variables. We assessed the cumulative incidence of epilepsy using the Kaplan-Meier method between the organophosphate poisoning and control cohorts. Their differences were estimated using a log-rank test. The incidence density rates (per 10,000 person years) by sex, age, and comorbidity were calculated for both cohorts. The relative hazard ratio (HR) of developing epilepsy in patients with organophosphate poisoning compared with patients without organophosphate poisoning was analyzed through univariable and multivariable Cox proportion hazard regressions. The multivariable model was adjusted for age, sex, and comorbidities of diabetes, hypertension, hyperlipidemia, head injury, depression, stroke, cirrhosis, CAD, CHF, and AF, simultaneously. Only confounding variables deemed significant in the multivariable model were further analyzed. Further analysis was performed to assess whether the association of epilepsy varied according to the length of the follow-up period after organophosphate poisoning was diagnosed. All analyses were conducted using the SAS statistical software package (Version 9.2 for Windows; SAS Institute, Inc., Cary, NC, USA). All statistical tests were performed considering a two-tailed significance level of 0.05.

## 3. Results

**Figure 1:**
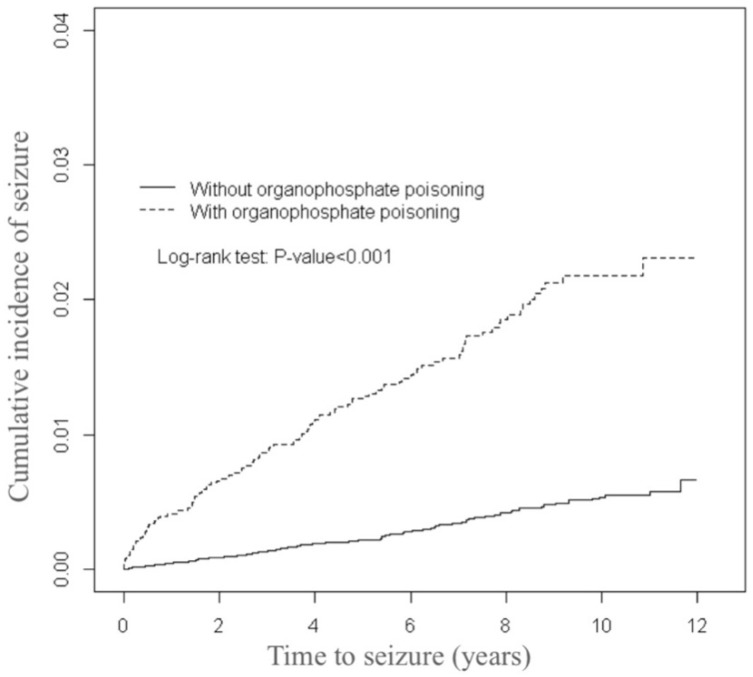
Comparison of the cumulative incidence of seizures among patients with and without organophosphate poisoning.

The distributions of the demographic variables and comorbidities for the organophosphate poisoning and control cohorts are presented in Table 1. The mean (±standard deviation (SD)) age of the organophosphate poisoning cohort was 53.3 (±16.1) years and that of the controls was 53.2 (±16.3) years, with 43.3% aged 20–49 years. Female patients were outnumbered by male patients. The organophosphate poisoning cohort was more likely to experience comorbidities than the control cohort (all *p*-values < 0.001). The mean follow-up times for the organophosphate poisoning and control cohorts were 5.50 (SD = 3.83) and 6.57 (SD = 3.42) years, respectively (data not presented). As illustrated by Figure 1, the cumulative incidence of seizures estimated through the Kaplan-Meier analysis was 1.63% higher in the organophosphate poisoning cohort than in the control cohort over the follow-up period (*p* < 0.001).

The overall incidence of epilepsy was 3.62-fold higher in the organophosphate poisoning cohort than in the control cohort (23.8 vs. 5.13 per 10,000 person years), with an adjusted HR of 3.57 (95% confidence interval (CI) = 2.73–4.68) during the 12 year follow-up period (Table 2). The gender-specific analysis revealed that the risk of epilepsy of the organophosphate poisoning cohort compared with that of the control cohort was significantly higher for both female (adjusted HR = 3.55, 95% CI = 1.93–6.52) and male patients (adjusted HR = 3.47, 95% CI = 2.56–4.69). The age-specific adjusted HR was highest in the patients with organophosphate poisoning aged 20–34 years (adjusted HR = 13.0, 95% CI = 5.40–31.4), followed by that of poisoning patients aged 35–49 years (adjusted HR = 5.61, 95% CI = 2.94–10.7), 50–64 years (adjusted HR = 2.19, 95% CI = 1.27–3.77), and 65 or older (adjusted HR = 2.48, 95% CI = 1.59–3.87). The comorbidity-specific analysis revealed that the incidence of epilepsy was higher in both cohorts among patients with comorbidities.

Table 3 depicts the interaction of organophosphate poisoning and comorbidities and its effect on the risk of epilepsy. Patients with organophosphate poisoning and stroke were at a much higher risk of seizures (adjusted HR = 15.1, 95% CI = 9.35–24.4) compared with patients with stroke alone (HR = 7.19, 95% CI = 4.58–11.3) or organophosphate poisoning alone (HR = 5.04, 95% CI = 3.80–6.68) (*p*-value of interaction = 0.004).

In addition, the interactions of organophosphate poisoning with hypertension interaction, head injury, CHF, and AF had significant effects on epilepsy risk (all interaction *p*-values < 0.05). When compared with individuals without organophosphate poisoning or cirrhosis, the adjusted HR increased to 16.6 (95% CI = 10.8–25.6) for patients with both organophosphate poisoning and cirrhosis. The risk decreased over time but persisted throughout the follow-up period (Table 4). The highest risk occurred during the first year of the follow-up period (adjusted HR = 7.71, 95% CI = 4.23–14.0) and decreased with time to 3.71 at 2–5 years of follow-up. The risk remained for more than 5 years of follow-up.

## 4. Discussion

To the best of our knowledge, this is the first report on the prevalence of seizure disorder in long-term outcomes after organophosphate poisoning. Compared with the control cohort, patients with organophosphate poisoning had a 3.57-fold higher seizure incidence (95% CI = 2.73–4.68). The patients with organophosphate poisoning with a history of stroke had a much higher risk of seizures than patients with stroke or organophosphate poisoning alone. The risk of seizures was highest during the first year of follow-up and decreased over the follow-up period, but remained significantly higher in the poisoning cohort after 5 years of follow-up.

The primary cause of death induced by organophosphate poisoning is respiratory paralysis; other major toxic signs include electrographic seizures in the brain and consequent motor convulsions. These seizures can rapidly progress to status epilepticus and can contribute to chronic brain damage [10]. Chronic brain damage can lead to the process of epileptogenesis [11]. Based on the available data for pesticides, seizures are more common in young patients. The incidence of tonic–clonic seizures averaged 25% in young patients, compared with 2.5% in adults [12]. Similar findings were observed in the present study. The risk of seizure disorder after organophosphate intoxication was higher in patients under 50 years of age. Previous reports have demonstrated that in young people, the predominant signs and symptoms associated with the central nervous system are common [12,13]. The incidence of tonic–clonic seizures is approximately 10-fold higher in young patients than in adults [14]. These age-related differences in the susceptibility to seizures can be attributed to differences in the dose to body weight ratio, metabolizing enzymes, permeability of membranes, and distribution of fat [12].

After any brain injury, subsequent seizures can occur due to the hyperexcitable electrical activity of the cerebral cortex after cell damage [15]. For example, patients with severe strokes or hemorrhagic strokes are at a higher risk of epileptic seizures. Galovic et al. developed a multivariate model evaluating the risk of late seizures within 5 years after a stroke [16]. Late seizures occurred in 4% of people during the first year after a stroke and in 8% within 5 years after a stroke. Roivainen et al. reported that patients from Finland aged 15–49 years had a 6.1% risk of late seizures in the first year and 9.5% within 5 years [17]. The first late seizure appeared more frequently within 1 year after a brain injury. Similar findings were observed in the present study of seizures in patients with organophosphate poisoning. The risk was highest during the first year, and the cumulative incidence of seizures remained elevated in the following years. Patients surviving organophosphate poisoning require a closer follow-up during the first year.

This study explored the risk of late-onset seizures after organophosphate poisoning. A higher percentage of seizures was observed in patients with comorbidities, for example in patients with a history of hypertension, stroke, head injury, CHF, or AF. A prospective, longitudinal, community-based cohort study examined the incidence and risk factors of seizures after an ischemic cerebral event (Framingham Heart Study) [18]. The study revealed that seizures occurred in 5.3% of participants after an ischemic cerebral event, and seizure incidence was 6.2% after cardioembolic events. AF and CHF are common conditions that predispose each other, share risk factors, and are associated with morbidity and mortality [19]. Irregular heartbeat and reduced cardiac output lead to reduced cerebral blood flow and potential brain damage [20,21,22]. Previous reports have indicated a relationship between AF and other neurological complications, such as seizure disorders [23,24]. This may suggest that the underlying risk factors and causes of brain lesions continue to expose patients to new seizure events.

## 5. Limitations

This study has several limitations. Firstly, because of its data-based study design, we were unable to control possible confounders (e.g., organophosphate poisoning severity, family history, medication, personal history), but the related comorbidities were measured reliably.

The second limitation of this study is that the severity and frequency of seizures could not be measured, and the seizure type could not be definitely classified in the analysis. Because electroencephalogram reports were not included in the data, patients with nonconvulsive seizures after organophosphate poisoning might have been underdiagnosed.

The third limitation of this study is that the diagnoses were provided by the treating physicians. The temporal relation between the onset of organophosphate exposure and seizures was based on the first appearance in the records. Therefore, the onset of the first seizure and the accurate diagnosis of epilepsy might have been considerably delayed.

The fourth limitation of this study is that the evidence derived from a retrospective record-based study is generally lower in statistical quality than that obtained from randomized trials. Despite a large-scale registry, meticulous study design, and the measures adopted to control confounding factors, bias resulting from unknown confounders may have affected our results.

Finally, patients with a history of comorbidities such as stroke, head injury, or atrial fibrillation have a higher risk of seizures. These comorbidities might influence the percentage of epilepsy in patients with organophosphate poisoning. Despite careful adjustment of related comorbidities, unknown confounders may have affected our results.

## 6. Conclusions

This population-based retrospective cohort study demonstrated that patients with a history of organophosphate poisoning have an increased long-term risk of seizures; furthermore, organophosphate poisoning may be an independent risk factor for seizures. These findings yield the following clinical implication: physicians should consider the history of exposure to organophosphates when making a new diagnosis of seizure, especially in young adults. Future mechanistic investigations of organophosphate exposure and the risk of PD are warranted.

## Figures and Tables

**Table 1:** Characteristics of patients with and without organophosphate poisoning.

Variable	Organophosphate Poisoning				*p*-Value
	Yes		No		
	(*N* = 9012)		(*N* = 36,048)		
	*N*	%	*N*	%	
Age, years					0.99
20–34	1331	(15.0)	5387	(14.5)	
35–49	2509	(28.3)	10,268	(27.7)	
50–64	2651	(29.9)	11,136	(30.0)	
≥65	2374	(26.8)	10,312	(27.8)	
Mean (SD) #	53.3	16.1	53.2	16.3	0.84
Gender					0.63
Female	2699	(30.5)	11,200	(30.2)	
Male	6166	(69.6)	25,903	(69.8)	
Comorbidity					
Diabetes	1170	(13.2)	1860	(5.01)	<0.001
Hypertension	1836	(20.7)	3112	(8.39)	<0.001
Hyperlipidemia	512	(5.78)	846	(2.28)	<0.001
Head injury	928	(10.5)	1060	(2.86)	<0.001
Depression	1477	(16.7)	158	(0.43)	<0.001
Stroke	717	(8.09)	1383	(3.73)	<0.001
Cirrhosis	755	(8.52)	892	(2.40)	<0.001
Coronary artery disease (CAD)	679	(7.66)	1456	(3.92)	<0.001
Congestive heart failure (CHF)	220	(2.48)	362	(0.98)	<0.001
Atrial fibrillation (AF)	120	(1.35)	275	(0.74)	<0.001

Chi-square test; #: Student’s t-test; SD denotes standard deviation.

**Table 2:** Incidence and hazard ratio of seizures for patients with and without organophosphate poisoning.

Variable	Organophosphate Poisoning							
	Yes			No				
Outcome	Event	PY	Rate ^#^	Event	PY	Rate ^#^	Crude HR ^†^ (95% CI)	Adjusted HR ^‡^
								(95% CI)
All	116	48,758	23.8	125	243,785	5.13	4.62 (3.59, 5.95) ***	3.57 (2.73, 4.68) ***
Gender								
Female	26	14,721	17.8	26	74,471	3.49	5.00 (2.90, 8.61) ***	3.55 (1.93, 6.52) ***
Male	90	34,037	26.4	99	169,314	5.85	4.51 (3.39, 6.01) ***	3.47 (2.56, 4.69) ***
Age, years								
20–34	24	8616	27.9	7	38,481	1.82	15.3 (6.57, 35.4) ***	13.0 (5.40, 31.4) ***
35–49	35	15,202	23.0	17	72,873	2.33	9.81 (5.50, 17.5) ***	5.61 (2.94, 10.7) ***
50–64	25	15,086	16.6	40	75,536	5.30	3.13 (1.90, 5.16) ***	2.19 (1.27, 3.77) **
≥65	32	9853	32.5	61	56,895	10.7	3.02 (1.97, 4.64) ***	2.48 (1.59, 3.87) ***
Comorbidity ^§^								
No	47	26,518	17.7	65	211,891	3.07	5.77 (3.97, 8.40) ***	5.93 (4.07, 8.63) ***
Yes	69	22,240	31.0	60	31,895	18.8	1.65 (1.17, 2.33) **	1.80 (1.25, 2.58) **

PY, person years; Rate ^#^, incidence rate per 10,000 person years. Crude HR ^†^, relative hazard ratio. Adjusted HR ^‡^, hazard ratio adjusted for age, sex, and comorbidities of diabetes, hypertension, hyperlipidemia, head injury, depression, stroke, cirrhosis, CAD, CHF, and AF. Comorbidity ^§^: Patients with any one of the comorbidities (including diabetes, hypertension, hyperlipidemia, head injury, depression, stroke, cirrhosis, CAD, CHF, and AF) were classified as the comorbidity group. ** *p* < 0.01, *** *p* < 0.001.

**Table 3:** Cox proportional hazard regression analysis of the risk of seizures associated with the interactions of organophosphate poisoning and comorbidities.

Variables		Crude HR ^†^ (95% CI)	Adjusted HR ^‡^ (95% CI)	*p*-Value ^†^
Organophosphate	Hypertension			<0.001
No	No	1 (Reference)	1 (Reference)	
No	Yes	6.32 (4.30–9.30) ***	4.99 (3.32–7.50) ***	
Yes	No	5.40 (4.01–7.28) ***	5.54 (4.11–7.47) ***	
Yes	Yes	10.0 (6.65–15.1) ***	8.84 (5.81–13.4) ***	
Organophosphate	Stroke			0.004
No	No	1 (Reference)	1 (Reference)	
No	Yes	9.28 (6.02–14.3) ***	7.19 (4.58–11.3) ***	
Yes	No	4.94 (3.72–6.54) ***	5.04 (3.80–6.68) ***	
Yes	Yes	17.7 (11.0–28.4) ***	15.1 (9.35–24.4) ***	
Organophosphate	Head injury			0.007
No	No	1 (Reference)	1 (Reference)	
No	Yes	5.27 (3.07–9.03) ***	4.96 (2.89–8.51) ***	
Yes	No	4.69 (3.57–6.17) ***	4.80 (3.65–6.32) ***	
Yes	Yes	9.07 (5.63–14.6) ***	9.50 (5.89–15.3) ***	
Organophosphate	Cirrhosis			0.96
No	No	1 (Reference)	1 (Reference)	
No	Yes	4.03 (2.04–7.94) ***	3.36 (1.70–6.63) ***	
Yes	No	4.09 (3.11–5.38) ***	4.19 (3.19–5.52) ***	
Yes	Yes	16.8 (10.9–25.9) ***	16.6 (10.8–25.6) ***	
Organophosphate	CHF			0.02
No	No	1 (Reference)	1 (Reference)	
No	Yes	13.6 (6.87–26.8) ***	9.86 (4.94–19.7) ***	
Yes	No	4.80 (3.70–6.23) ***	4.92 (3.79–6.38) ***	
Yes	Yes	16.5 (6.72–40.3) ***	13.6 (5.53–33.5) ***	
Organophosphate	AF			0.04
No	No	1 (Reference)	1 (Reference)	
No	Yes	12.9 (6.01–27.7) ***	9.01 (4.16–19.5) ***	
Yes	No	4.75 (3.67–6.15) ***	4.88 (3.76–6.32) ***	
Yes	Yes	16.7 (6.15–45.2) ***	13.1 (4.81–35.6) ***	

Crude HR ^†^, relative hazard ratio; Adjusted HR ^‡^, hazard ratio adjusted for age and sex. *** *p* < 0.001. ^†^ *p* for interaction.

**Table 4:** Trends of seizure event risk stratified by follow-up years.

Variables	Organophosphate Poisoning							
	Yes			No				
Follow-Up Time, years	Event	PY	Rate	Event	PY	Rate	Crude HR ^†^ (95% CI)	Adjusted HR ^‡^ (95% CI)
≤1	32	7521	42.6	18	35,870	5.02	8.39 (4.71–14.9) ***	7.71 (4.23–14.0) ***
2–5	52	23,939	21.7	51	118,635	4.30	5.05 (3.43–7.43) ***	3.71 (2.45–5.62) ***
>5	32	17,298	18.5	56	89,280	6.27	2.95 (1.91–4.55) ***	2.13 (1.33–3.41) **

PY, person years; Rate, incidence rate per 10,000 person years; Crude HR ^†^, relative hazard ratio; Adjusted HR ^‡^, hazard ratio adjusted for age, sex, and comorbidities of diabetes, hypertension, hyperlipidemia, head injury, depression, stroke, cirrhosis, CAD, CHF, and AF. ** *p* < 0.01, *** *p* < 0.001.

## References

[1] Peter J.V., Sudarsan T.I., Moran J.L. (2014). Clinical features of organophosphate poisoning: A review of different classification systems and approaches. Indian J. Crit. Care. Med..

[2] Indira M., Andrews M.A., Rakesh T.P. (2013). Incidence, predictors, and outcome of intermediate syndrome in cholinergic insecticide poisoning: A prospective observational cohort study. Clin. Toxicol..

[3] Eddleston M., Mohamed F., Davies J.O., Eyer P., Worek F., Sheriff M.H., Buckley N.A. (2006). Respiratory failure in acute organophosphorus pesticide self-poisoning. Mon. J. Assoc. Physicians.

[4] Wu X., Kuruba R., Reddy D.S. (2018). Midazolam-resistant seizures and brain injury after acute intoxication of diisopropylfluorophosphate, an organophosphate pesticide and surrogate for nerve agents. J. Pharmacol. Exp. Ther..

[5] Hobson B.A., Rowland D.J., Supasai S., Harvey D.J., Lein P.J., Garbow J.R. (2018). A magnetic resonance imaging study of early brain injury in a rat model of acute dfp intoxication. Neurotoxicology.

[6] Scholl E.A., Miller-Smith S.M., Bealer S.L., Lehmkuhle M.J., Ekstrand J.J., Dudek F.E., McDonough J.H. (2018). Age-dependent behaviors, seizure severity and neuronal damage in response to nerve agents or the organophosphate dfp in immature and adult rats. Neurotoxicology.

[7] Meletti S., Lucchi C., Monti G., Giovannini G., Bedin R., Trenti T., Rustichelli C., Biagini G. (2018). Low levels of progesterone and derivatives in cerebrospinal fluid of patients affected by status epilepticus. J. Neurochem..

[8] Flannery B.M., Bruun D.A., Rowland D.J., Banks C.N., Austin A.T., Kukis D.L., Li Y., Ford B.D., Tancredi D.J., Silverman J.L. (2016). Persistent neuroinflammation and cognitive impairment in a rat model of acute diisopropylfluorophosphate intoxication. J. Neuroinflammation.

[9] Siso S., Hobson B.A., Harvey D.J., Bruun D.A., Rowland D.J., Garbow J.R., Lein P.J. (2017). Editor’s highlight: Spatiotemporal progression and remission of lesions in the rat brain following acute intoxication with diisopropylfluorophosphate. Toxicol. Sci..

[10] Shrot S., Ramaty E., Biala Y., Bar-Klein G., Daninos M., Kamintsky L., Makarovsky I., Statlender L., Rosman Y., Krivoy A. (2014). Prevention of organophosphate-induced chronic epilepsy by early benzodiazepine treatment. Toxicology.

[11] Curia G., Lucchi C., Vinet J., Gualtieri F., Marinelli C., Torsello A., Costantino L., Biagini G. (2014). Pathophysiogenesis of mesial temporal lobe epilepsy: Is prevention of damage antiepileptogenic?. Curr. Med. Chem..

[12] Karalliedde L.D., Edwards P., Marrs T.C. (2003). Variables influencing the toxic response to organophosphates in humans. Food. Chem. Toxicol..

[13] Lifshitz M., Shahak E., Bolotin A., Sofer S. (1997). Carbamate poisoning in early childhood and in adults. Clin. Toxicol..

[14] Tattersall J. (2009). Seizure activity post organophosphate exposure. Front. Biosci. (Landmark Ed.).

[15] Avoli M., D’Antuono M., Louvel J., Kohling R., Biagini G., Pumain R., D’Arcangelo G., Tancredi V. (2002). Network and pharmacological mechanisms leading to epileptiform synchronization in the limbic system in vitro. Prog. Neurobiol..

[16] Galovic M., Dohler N., Erdelyi-Canavese B., Felbecker A., Siebel P., Conrad J., Evers S., Winklehner M., von Oertzen T.J., Haring H.P. (2018). Prediction of late seizures after ischaemic stroke with a novel prognostic model (the select score): A multivariable prediction model development and validation study. Lancet. Neurol..

[17] Roivainen R., Haapaniemi E., Putaala J., Kaste M., Tatlisumak T. (2013). Young adult ischaemic stroke related acute symptomatic and late seizures: Risk factors. Eur. J. Neurol..

[18] Stefanidou M., Das R.R., Beiser A.S., Sundar B., Kelly-Hayes M., Kase C.S., Devinsky O., Seshadri S., Friedman D. (2017). Incidence of seizures following initial ischemic stroke in a community-based cohort: The framingham heart study. Seizure.

[19] Wang T.J., Larson M.G., Levy D., Vasan R.S., Leip E.P., Wolf P.A., D’Agostino R.B., Murabito J.M., Kannel W.B., Benjamin E.J. (2003). Temporal relations of atrial fibrillation and congestive heart failure and their joint influence on mortality: The framingham heart study. Circulation.

[20] Udompanich S., Lip G.Y., Apostolakis S., Lane D.A. (2013). Atrial fibrillation as a risk factor for cognitive impairment: A semi-systematic review. Mon. J. Assoc. Physicians.

[21] Gaita F., Corsinovi L., Anselmino M., Raimondo C., Pianelli M., Toso E., Bergamasco L., Boffano C., Valentini M.C., Cesarani F. (2013). Prevalence of silent cerebral ischemia in paroxysmal and persistent atrial fibrillation and correlation with cognitive function. J. Am. Coll. Cardiol..

[22] Hsu C.Y., Chen T.H., Su Y.W., Chang C.C., Chen M.H., Leu H.B., Huang P.H., Chen J.W., Lin S.J. (2016). Usefulness of the chads2 score for determining risk of seizure in patients with atrial fibrillation. J. Am. Coll. Cardiol..

[23] Herskovitz M., Schiller Y. (2012). Atrial fibrillation associated with epileptic seizures. Arch. Neurol..

[24] Gardezi S.K., Chalmers R.M., Pegge N.C. (2014). Seizure secondary to cardiac arrythmias. Scott. Med. J..

